# Cerebral palsy: a comprehensive review

**DOI:** 10.1186/1753-6561-9-S3-A70

**Published:** 2015-05-19

**Authors:** Caroline Leclercq

**Affiliations:** 1 Institut de la Main, 75016, Paris, France

## 

Cerebral palsy (CP) includes all the sequelae of infantile encephalopathies occurring during the perinatal period or during infancy. The most common deformity is *infantile cerebral hemiplegia*, characterised by spasticity associated with various degrees of motor and sensory deficit in the ipsilateral upper and lower limbs. It manifests progressively during growth, but once established, follows a non-progressive course, which makes it amenable to surgical treatment.

Clinical examination is the key to successful treatment. It is best performed with all the specialists involved in the child’s care. It is performed with a fourfold goal:

• Evaluate spasticity.

• Evaluate any secondary muscle and/or joint contracture, which may require a specific treatment

• Evaluate the motor and sensory deficit in the upper limb.

• Assess the existing function and functional needs of the upper limb.

Symptoms may vary with the child’s emotional state and fatigue level. Repeated examination and video recording are most helpful, both in the decision-making and in the evaluation of treatment outcome.

Spasticity is rarely an isolated feature. A number of other neurological and orthopaedic impairments may be associated. They also need to be carefully assessed before planning any treatment.

Surgery has a limited place in the treatment of spasticity of the upper limb. It is only one element of the rehabilitative care, which includes primarily physiotherapy and splinting, occupational therapy, and pharmacological treatment as needed. Botulinum toxin has a special place in this armamentum, effective both in the diagnosis and in the treatment of sapsticity. We use it routinely before any surgical decision. Any decision-making should include the patient and his/her family, as well as all the physicians and care-givers involved in the treatment.

Surgery in CP patients seems more effective if performed earlier in the patient’s life, preferably during childhood. There is some evidence that improved use of an extremity can improve cortical representation of the extremity and hopefully decrease the development of neglect. Otherwise, the growing child develops some ‘tricks’ with the contralateral healthy limb, which often prove difficult to overcome or reverse after surgery, rendering the results of surgical treatment much less functional.

Surgery aims at:

• Reducing spasticity (selective neurectomy)

• Relieving muscle / joint contracture (muscle / tendon lengthening, arthrolysis)

• Augmenting paralysed muscles, whenever possible by tendon transfers.

All these procedures are preferably performed during a single operative session, in order to restore the balance between hypertonic flexor/pronator muscles, and paralysed or pseudo-paralysed extensor/ supinator muscles

Ideally, surgery is undertaken in order to improve function of upper limb. In that perspective, “the ideal candidate is a cooperative 6-year-old child, with stable family support, who has a predominantly spastic upper limb deformity, with satisfactory hand sensibility, hemiplegic or monoplegic and without significant neurological deficits” (Tonkin). But in other groups of patients, surgery can be very helpful in relieving pain, facilitating nursing, or improving cosmesis.

